# Direct observation of ion emission from charged aqueous nanodrops: effects on gaseous macromolecular charging†

**DOI:** 10.1039/d0sc05707j

**Published:** 2021-02-27

**Authors:** Conner C. Harper, Daniel D. Brauer, Matthew B. Francis, Evan R. Williams

**Affiliations:** Department of Chemistry, University of California Berkeley California 94720-1460 USA erw@berkeley.edu

## Abstract

Mechanistic information about how gaseous ions are formed from charged droplets has been difficult to establish because direct observation of nanodrops in a size range relevant to gaseous macromolecular ion formation by optical or traditional mass spectrometry methods is challenging owing to their small size and heterogeneity. Here, the mass and charge of individual aqueous nanodrops between 1–10 MDa (15–32 nm diameter) with ∼50–300 charges are dynamically monitored for 1 s using charge detection mass spectrometry. Discrete losses of minimally solvated singly charged ions occur, marking the first direct observation of ion emission from aqueous nanodrops in late stages of droplet evaporation relevant to macromolecular ion formation in native mass spectrometry. Nanodrop charge depends on the identity of constituent ions, with pure water nanodrops charged slightly above the Rayleigh limit and aqueous solutions containing alkali metal ions charged progressively below the Rayleigh limit with increasing cation size. MS2 capsid ions (∼3.5 MDa; ∼27 nm diameter) are more highly charged from aqueous ammonium acetate than from its biochemically preferred, 100 mM NaCl/10 mM Na phosphate solution, consistent with ion emission reducing the nanodrop and resulting capsid charge. The extent of charging indicates that the capsid partially collapses inside the nanodrops prior to the charging and formation of the dehydrated gaseous ions. These results demonstrate that ion emission can affect macromolecular charging and that conformational changes to macromolecular structure can occur in nanodrops prior to the formation of naked gaseous ions.

## Abstract 2

Ion evaporation from aqueous nanodrops is measured for the first time using charge detection mass spectrometry, and the origin of solute ion dependent charging of large (MDa) macromolecules is revealed.

## Graphical abstract

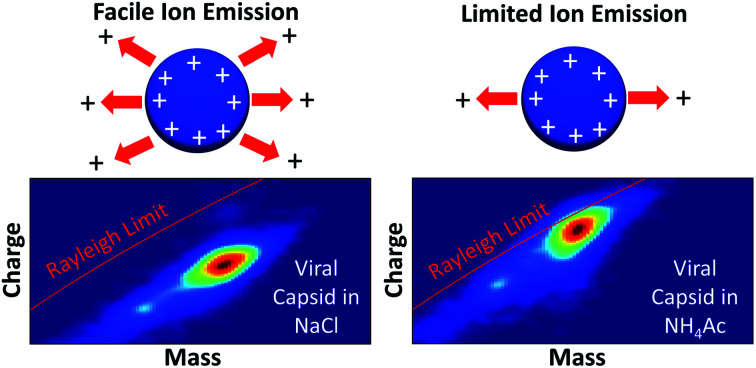

## Introduction

Electrospray ionization (ESI) is used in thousands of laboratories worldwide to produce ions directly from solution for analysis by mass spectrometry (MS), yet the mechanism(s) by which gaseous ions are generated has been subject to conjecture and much debate.^1–5^ Charged droplets initially generated by electrospray undergo solvent evaporation, which increases the strength of the electric field at the droplet surface. This can lead to droplet fission when the charge–charge repulsion exceeds the force of surface tension holding the droplet intact. Droplet instability occurs when the net charge on a droplet approaches the Rayleigh limit (*q*_R_), which results in the emission of smaller charged droplets from the surface.^6^

1 *q*_R_ = 8π(*ε*_0_*γR*^3^)^1/2^

where *γ* is the surface tension, *q*_R_ is the critical net charge on a spherical droplet with a radius, *R*, and *ε*_0_ is the permittivity of free space. The Rayleigh fission process has been investigated for large droplets (>10's of μm) using a variety of experimental apparatus.^7–9^ Leisner and coworkers used high-speed microscopy to show the Rayleigh induced breakup of ∼24 μm ethylene glycol droplets, which resulted in the formation of ∼100 progeny droplets that carry away ∼33% of the charge of the original droplet, but only 0.3% of its mass.^7^ Successive fission events of individual charged droplets between 5–40 μm were measured using phase Doppler interferometry combined with a drift field reversing “ping-pong” technique.^8^ Fission of small water droplets (5–20 μm) occurred near the Rayleigh limit whereas acetonitrile and methanol droplets charged up to 120% of the Rayleigh limit prior to fission.^8^

Observing discharge events for smaller “nanodrops” (10–100's of nm) is more difficult than for μm-sized droplets because of the challenge associated with using optical methods with droplets that are smaller than the wavelengths of light. Nanodrops containing one or more ions with up to ∼600 water molecules, corresponding to diameters <2 nm, have been trapped, mass analyzed, and probed using infrared photodissociation spectroscopy and ultraviolet photodissociation to obtain information about ion–water interactions^10–12^ and water molecule binding energies.^13^ Charge separation processes can occur in these small nanodrops that contain multivalent ions when sequential evaporation of water molecules reduces the cluster size to where the multivalent ion is no longer stable.^12–15^ For example, La^3+^(H_2_O)_*n*_ undergoes charge separation to form H^+^(H_2_O)_*x*_ and LaOH^2+^(H_2_O)_*y*_ when *n* < 19.^15^ Transitions between conformations of small peptides in solution to their stable gaseous ion conformations have been measured with ion mobility of hydrated ions.^16^ These cluster studies provide information about processes that occur very late in the dehydration stages of gaseous ions formed by electrospray. However, there remains a significant gap in nanodrop size (2–1000 nm) for which direct experimental observations have not previously been possible.

Because of the inability to directly monitor what occurs inside aqueous nanodrops between 2 and 1000 nm, much of what is thought about how gaseous ions are formed has been inferred indirectly from a number of different measurements and from theory. Most mechanisms for dehydrated gaseous ion formation are variations on two long-standing models: the ion evaporation model (IEM)^17^ and the charge residue model (CRM).^18^ In the CRM, solvent evaporation occurs from small droplets that contain one or more analyte molecules, and the analyte charge is limited by the charge on the droplet.^18^ This mechanism is widely invoked in native mass spectrometry where charging of large proteins and macromolecular complexes follows a trend in charging that is limited by the Rayleigh charge on a droplet of similar size to that of the analyte.^3^ Charging of analyte molecules that cannot change conformation follows this trend for solvents with different surface tensions.^19^ In the IEM, ions are ejected from the surface of small droplets (∼10 nm) with only a limited number of solvent molecules when the electric field at the surface is sufficiently high.^17^ The IEM has been widely invoked for the formation of small ionic species.^2,5,17^ Ion emission events are characterized by the ejection of ions that carry only a small number of solvent molecules in addition to the charge carrier.^5,17,20^ Less strongly solvated ions are more readily emitted from a droplet, consistent with formation of more abundant gaseous alkali metal cations and halide anions with increasing ion size.^21^

Formation of gaseous ions from small highly charged nanodrops has been investigated computationally.^4,5,20,22–24^ Molecular dynamics (MD) simulations support ion evaporation of Na^+^ and other small ions from nanodrops with diameters <5.5 nm.^22–24^ For droplets that contain larger analytes, results from MD simulations suggest the possibility of several different mechanisms. Simulations by Consta and co-workers indicate that droplets that contain a DNA or RNA ion can distort into “star” morphologies in the late stages of desiccation.^23^ Some simulations suggest that part of an unfolded or unfolding macromolecule at the surface of the droplet is extruded into the gas phase (an IEM-like process) followed by a stepwise ejection of the remainder of the molecule as a highly charged ion.^4,5^ This process is analogous to the process for asymmetric charge partitioning typically observed for the collision induced dissociation of large macromolecular complexes.^25,26^ Recent simulations by Aliyari and Konermann suggest an ion emission-like process may also occur for small native proteins that carry a sufficiently high charge in solution.^24^

Following on early pioneering work by Gamero-Castaño and Fernández de la Mora,^27^ Hogan *et al.*^28^ proposed a hybrid model that combines both the charge residue and field emission models (combined charge residue and field emission model or CCRFEM). In this model, the charge states of proteins and other macromolecules are not determined solely by the Rayleigh limit of a droplet of similar size, as would the be case for just the CRM, but rather the charge is also limited by field-induced emission of small charged solute ions and clusters from the protein-containing nanodroplet (an IEM process).^28^ Ions that are not as strongly solvated are more easily emitted from the droplet and leave less charge available for the protein. A tandem differential mobility analysis of sub-micrometer particles indicates a transition from Rayleigh fission-controlled to ion emission-controlled discharging occurs for methanol–water nanodrops at ∼40 nm.^29^ The mean charge state of many proteins is reduced when these ions are formed from solutions containing small ions that have low solvation energies consistent with charge reduction *via* ion evaporation.^28,30,31^ However, there was no significant difference in the charging of several proteins with different pI values from solutions containing different alkali metal acetates, suggesting that ions with widely different solvation energies do not necessarily affect protein charging.^32^ A complication with these experiments is that protein conformation, which has a significant effect on protein charging, can differ in solutions with different ionic constituents, even when the ionic strength and pH of the solutions are the same.^33^ There have been no direct experimental observations of ion emission from nanodrops in the size range (∼10–40 nm) where ion emission is thought to play a role in the charging of macromolecules in the CCRFEM.

Charge detection mass spectrometry (CDMS) can weigh individual ions well into the 100's of megadaltons (MDa) corresponding to molecules or molecular assemblies with diameters over 100 nm.^34–40^ Sample heterogeneity and salt adduction can lead to overlapping ion signals that can prevent conventional mass spectrometry measurements of ion ensembles.^41,42^ This problem with high sample heterogeneity is overcome with CDMS measurements of individual ions, making it an ideal technique for probing charged nanodrops. These measurements can serve as a bridge between what is known about micron-sized droplet charging and fission and events that occur for nanodrops closer to the size of typical analyte molecules.

Here, aqueous nanodrops ranging in mass from ∼1–10 MDa (∼15–32 nm diameter) are investigated using CDMS. The nanodrop charge and mass depends on the identity of salts in solution, and these values change over the one second trapping time, leading to the first direct observation of ion emission from nanodrops in a size range representing late stages of droplet evolution prior to formation of gaseous ions of proteins and macromolecular complexes. Differences in charging of the bacteriophage MS2 capsid formed from aqueous ammonium acetate *versus* its biochemically preferred sodium-containing buffer solution indicate that solute ion identity affects the final charge states of this complex through ion evaporation of small ions. Moreover, the extent of charging indicates that the structure of the capsid collapses in the nanodrop prior to gaseous ion formation.

## Experimental

### Charge detection mass spectrometry

All mass and charge data for individual ions were obtained using a home-built charge detection mass spectrometer.^36,37^ Nanoelectrospray ionization using borosilicate capillaries (1.0 mm outer diameter, 0.78 mm inner diameter, Sutter Instruments, Novato, CA) with tips that are pulled to an inner diameter of ∼1.5 μm using a Flaming/Brown P-87 micropipette puller (Sutter Instruments, Novato, CA) is used to introduce ions into the instrument through a modified Waters Z-Spray source (Waters Corporation, Milford, MA). Ions enter a region containing two RF quadrupole ion guides (Ardara Technologies, Ardara, PA) and are accumulated in the second ion guide for up to 1 s. They are pulsed into the electrostatic ion trap at a pressure of ∼4 × 10^−9^ torr where they are stored for 1 s. A charge pulse is induced each time an ion traverses the detector tube. Signals are analyzed using short-time Fourier transforms (STFT)^43^ using a 25 ms segment length stepped across the transient in 5 ms increments. This segment length was chosen to minimize amplitude dampening that is caused by ion frequency shifts with time.^44^ Data for only those ions trapped and detected for the entire 1 s trapping period (>70% of all ions) were analyzed. Individual ion masses, charges, and energies are dynamically determined from the measured oscillation frequencies and amplitudes of the fundamental and second harmonic frequencies^45^ as they evolve throughout the 1 s trapping period. The broad distribution of ion mass and charge in these experiments makes it possible to simultaneously analyze up to ∼40 individual ions in each trapping event because the frequency distribution of the ions is broad and their signals are less likely to interfere with each other.^43,44^ Additional experimental details are provided in the ESI.† Aqueous solutions of ammonium acetate (AA), LiCl, NaCl, KCl and CsCl at 20 mM salt solutions were prepared using Millipore Milli-Q water.

### MS2 capsid synthesis

Bacteriophage MS2 virus-like particles were produced as previously described.^46^ In brief, DH10B *E. coli* containing a pBAD MS2 expression vector (chloramphenicol resistant) was grown to OD0.6 in 2xYT media at 37 °C. Expression was induced *via* addition of arabinose to 0.1% (w/v) and cultures were incubated for 18 h at 37 °C. Cells were then harvested *via* centrifugation and lysed by sonication. The collected lysate was purified through two rounds of ammonium sulfate precipitation at 50% saturation followed by polishing on an ÄKTA start FPLC system using a prepacked Sephacryl S-500 HR column. The resultant purified capsids were buffer exchanged into the desired buffers using a 100 kDa MWCO spin concentrator. Details about the small angle X-ray scattering (SAXS) and dynamic light scattering (DLS) experiments performed on MS2 solutions are provided in the ESI.†

## Results and discussion

### Acquiring individual nanodrop data

The mass and charge of individual nanodrops generated by electrospray ionization from solutions consisting of either pure water or aqueous solutions with various salts at a concentration of 20 mM were measured using CDMS. Data were acquired with “soft” ESI conditions where the voltage difference between skimmer cones (∼5 torr region) is low (∼35 V) to minimize collisional activation. Ions are trapped in a quadrupole ion guide for between 0–1 s prior to introduction into the electrostatic ion trap for 1 s. The resulting nanodrop lifetimes are between ∼1 and 2 s during which time the frequency and intensity of each ion is measured. The energy of each ion throughout the measurement is obtained from the ratio of the amplitude of the 2^nd^ harmonic frequency to that of the fundamental frequency.^45^ The *m*/*z* of each ion is obtained from its energy and frequency, and the charge is obtained from the amplitude of the signal.^45,47^ Thus, the mass, charge, and energy of every ion is measured and tracked throughout the 1 s trapping time. The data from many such trapping events are compiled until a statistically robust number of ions (typically several thousand) are measured.

**Fig. 1:**
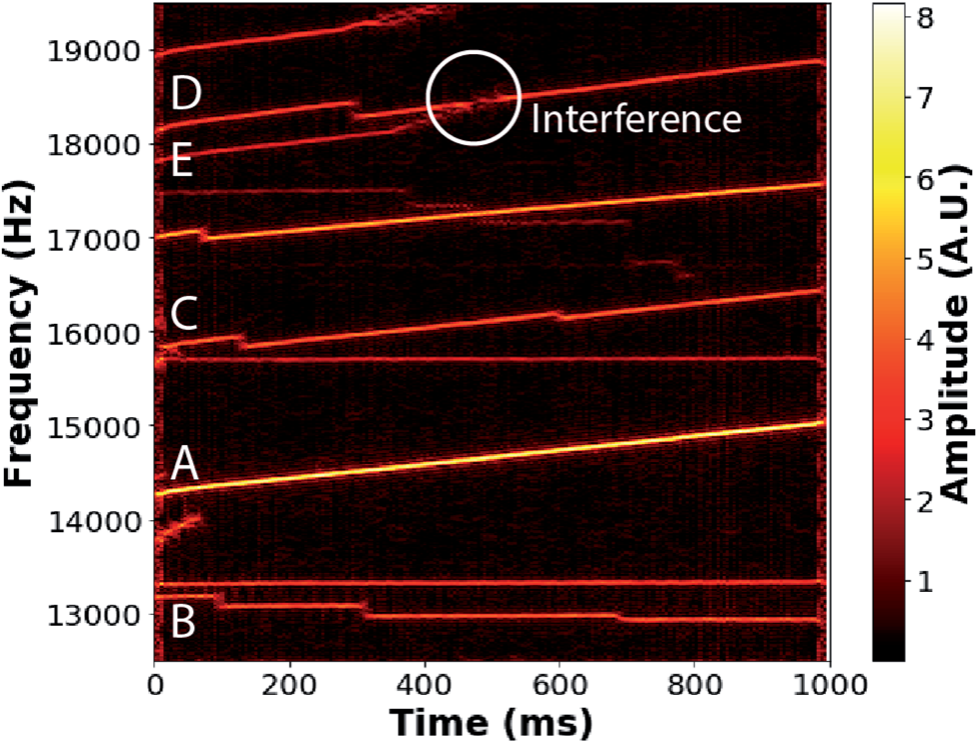
Short-time Fourier transform of the time-domain signal from a 1 s trapping event showing the frequency evolution of 11 ions originating from a 20 mM NaCl solution as a function of trap time. Seven ions are trapped for the entire 1 s trapping time. Increases in ion frequency, *e.g.*, for ions labeled A, C, D, and E, correspond to decreasing ion energies, primarily as a result of water evaporation from the ions. Discrete, negative changes in frequency observed for ions B, C, and D correspond to charge losses. Any ion frequencies that overlap during the trapping period, such as ions D and E, are discarded from the analysis as inaccurate/indistinguishable amplitudes (white circle) occur as a result of interference.

An example of short-time Fourier transform (STFT) data obtained from a single trapping event for ions produced from a 20 mM solution of NaCl is shown in Fig. 1. This STFT analysis method makes it possible to obtain the evolution of the ion frequency (*y*-axis) and amplitude (color scale) over the trapping period (*x*-axis) with a time resolution of 5 ms corresponding to the STFT step size. Several individual ion signals, corresponding to contiguous lines, persist over the acquisition time. The signal amplitudes are related to the ion charge,^47^ and the ion frequencies (*f*) are related to the *m*/*z* of ions *via*eqn (2):2
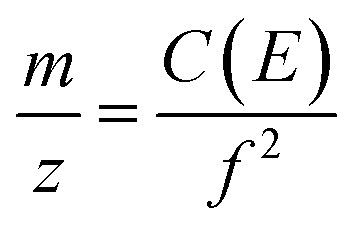
where *C*(*E*) is a function of ion energy per charge.^37,45^ Ion frequencies increase, *e.g.*, A and C in Fig. 1, as a result of both collisional energy losses to the background gas and energy lost as a result of neutral mass losses, *i.e.*, water evaporation from the nanodrops. Discrete, negative frequency changes occur as a result of sudden charge loss, which increases the *m*/*z* of the remaining nanodrop. Examples of these charge losses can be seen in ion signals B, C, and D in Fig. 1. Ion signals may occasionally overlap and interfere in frequency space, as occurs for ion signals D and E in Fig. 1. Data from such interfering ions are not used in subsequent analysis.

The two pathways for energy loss, collisions with background gas and neutral mass losses, can be deconvolved, making it possible to measure the neutral mass that is lost from each nanodrop during the trapping event.^45^ The ranges of mass loss from the initially trapped ions can vary widely depending on the solution composition, but some of the initially trapped ions lose 100's of kDa. Significant mass is lost from pure aqueous nanodrops, indicating that evaporation of up to many thousands of water molecules occurs. Such high evaporative losses from ions are not typically observed in CDMS experiments because greater initial ion activation is commonly used to desolvate ions when measuring the masses of large macromolecules.

### Nanodrop mass and charge distributions from different aqueous solutions

**Fig. 2:**
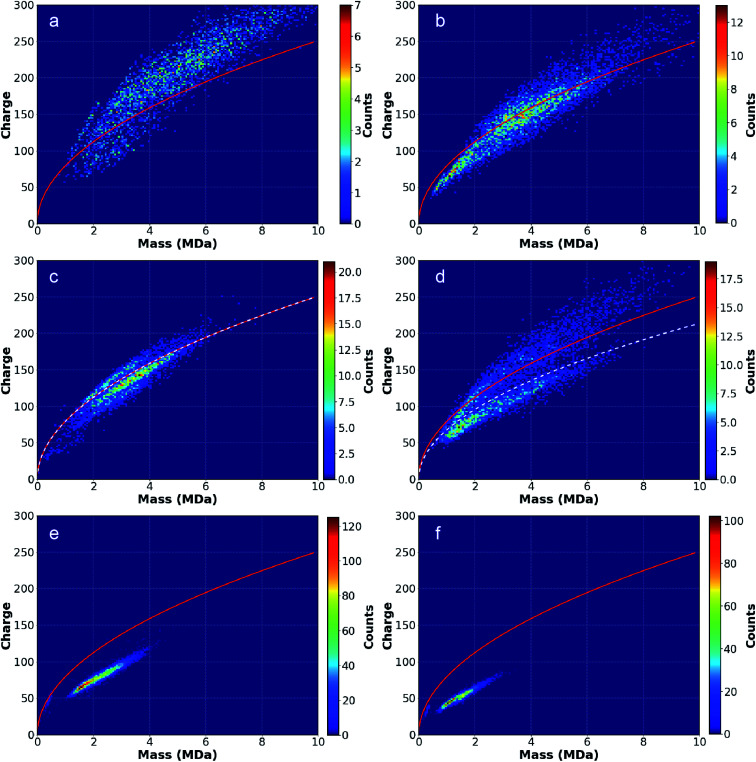
Two-dimensional mass *vs.* charge histograms for ions originating from (a) pure water, (b) AA, (c) LiCl, (d) NaCl, (e) KCl and (f) CsCl. Red lines indicate the Rayleigh limit charge as function of mass calculated for water with a density of 0.998 g mL^−1^. White dotted lines in the LiCl and NaCl data distinguish “wet”, higher charge distributions of ions from “dry”, lower charge distributions. Higher mass “wet” ions present in (a)–(d) have higher charge relative to the Rayleigh limit than lower mass “wet” ions, suggesting a progression toward a “dry” state at lower mass. “Dry” ions, observed in (c)–(f), have charges below the Rayleigh limit that decrease as a function of increasing alkali metal cation size.

Two-dimensional mass *vs.* charge histograms for ions formed from solutions of pure H_2_O and aqueous solutions containing AA, LiCl, NaCl, KCl, and CsCl, each at 20 mM, are shown in Fig. 2a–f, respectively. The red lines correspond to the Rayleigh charge limit for a pure aqueous spherical droplet calculated using the bulk surface tension (0.07286 N m^−1^ at 20 °C) and the density (0.998 g mL^−1^ at 20 °C) of water. The number of ions in each histogram bin, corresponding to a small range of mass and charge, is represented with a color scale. Many charged nanodrops lose significant mass and/or charge (Fig. 1) over the trapping period. The average mass and charge of each ion over this period were used in these histograms.

The charged nanodrops formed from pure H_2_O (Fig. 2a) have the largest average mass and charge. The average mass and charge from AA (Fig. 2b) are about 1 MDa and 50 elementary charges (*e*) lower. The mass and charge continue trending downward for the alkali metal ions with increasing cation size (Fig. 2c–f). Ions produced from aqueous CsCl have on average ∼25% of the mass and charge of the pure aqueous nanodrops. The relationship between nanodrop mass and solute ion identity is discussed in the ESI.† The charge of each ion was divided by the Rayleigh charge limit for an aqueous droplet (red line) for an ion of that mass to give the ion charge as a percentage of the expected Rayleigh charge (Table 1). Notably, ions formed from pure water are on average charged well above the computed Rayleigh limit (118%) whereas ions from all other solutions are typically charged below the limit (∼56% with CsCl). For H_2_O and AA, higher mass nanodrops (>3 MDa) tend to be more highly charged relative to the Rayleigh limit compared to lower mass ions from the same solution, perhaps as a result of deviations in surface tension from bulk values at smaller nanodrop size. There is also a strong dependence between alkali metal ion size and charging relative to the Rayleigh limit, with the LiCl solution having the highest average charge relative to the Rayleigh limit (96%) and CsCl the lowest (56%).

**Table 1:** Physical properties of nanodrops originating from solutions containing different solutes

	Pure H_2_O	AA	LiCl (all)	LiCl (“dry”)	LiCl (“wet”)	NaCl (all)	NaCl (“dry”)	NaCl (“wet”)	KCl	CsCl
Ion count	3523	5861	4322	3181	1141	5616	2597	3019	8255	3078
Avg. mass (kDa)	5118	3992	3106	3009	3378	3343	2890	3733	2006	1352
Avg. mass loss (kDa)	319	189	30	18	68	71	18	145	9	5
Avg. charge	207.0	152.8	129.5	121.2	152.5	132	101.5	158.2	73.9	51.3
Avg. % of Rayleigh charge	118%	99%	96%	92%	106%	94%	78%	106%	69%	56%
Ion emission rate (events per ion)	0.228	0.189	0.527	0.475	0.708	0.305	0.365	0.277	0.006	0.009

Interpretation of data for nanodrops formed from LiCl and NaCl solutions is complicated by the presence of two distinct distributions of ions with overlapping masses but with different charges. Ions in one distribution are charged below the Rayleigh limit whereas ions in the other distribution are charged slightly above the Rayleigh limit. Due to these differences, these ion distributions in the LiCl and NaCl data were analyzed separately. Data for NaCl obtained under more typical activating conditions resulted in only the lower charge distribution. Based on these and other data, the higher charge distribution for LiCl and NaCl are referred to as “wet” whereas the lower charge distribution is referred to as “dry”. The “wet” LiCl and NaCl ion distributions follow the trend of greater deviation from the Rayleigh limit with greater mass noted for H_2_O and AA. In contrast, the lower charge, “dry” distributions are more qualitatively similar to the KCl and CsCl ion distributions.

### Comparing individual “dry” and “wet” ions

**Fig. 3:**
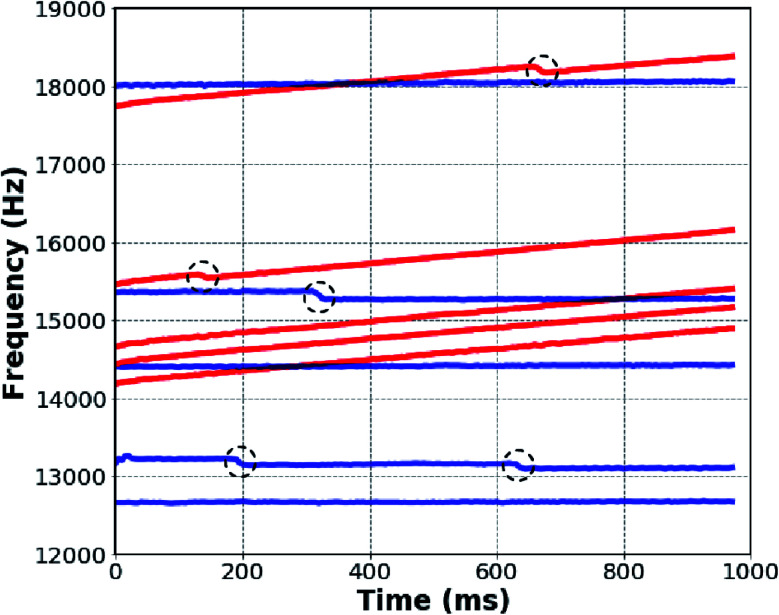
Ion frequency *vs.* trap time for five “dry” (blue) and five “wet” (red) individual ions randomly selected from the two distributions observed in NaCl solution data of Fig. 2D. Ion emission events (black circles) occur with both “dry” and “wet” ions. The frequencies of “wet” ions increase significantly, primarily as a result of neutral mass losses of 100's of kDa. The frequency increase of “dry” ions occurs less rapidly.

Because CDMS measurements are made on an ion-by-ion basis, analysis of individual ion signals can provide additional insights into the overall analyses of ion ensembles. For ion ensembles containing more than one distribution of ions, such as LiCl and NaCl, this type of analysis is especially informative because different physical traits of ions that make up the two distinct “wet” and “dry” ion distributions can be distinguished. Five individual ions from both the “wet” and “dry” distributions from NaCl were randomly selected from different trapping events, and the STFT ion peak amplitude traces are shown together in Fig. 3. Blue traces correspond to the “dry” individual ions below the white dotted line of Fig. 2D; red traces correspond to “wet” ions above the line. The frequencies of ions from the “dry” distribution (blue) remain relatively constant, indicating that the *m*/*z* values and ion energies do not change significantly over the trapping period. However, some abrupt drops in frequency that correspond to charge losses occur (circled in black in Fig. 3). Although a sample of only five ions is shown in Fig. 3, nearly all ions in the lower charge distribution exhibit similar behavior. Ions from the higher charge “wet” distribution (red) also show abrupt frequency drops (black circles), but in contrast with the ions in the lower charge “dry” distribution, the frequency of these ions increases significantly at all other times. The frequencies of these ions continuously increase as a result of neutral mass losses on the order of ∼100 kDa (∼5600 water molecules). Mass loss data for each solution as well as the “wet” and “dry” distributions observed for LiCl and NaCl are included in Table 1. Nearly all ions in the upper charge distribution undergo more extensive mass loss than ions in the lower distribution. This individual ion analysis provides additional support for the classification of the two distributions as “wet” and “dry” ions with notably different physical characteristics. Although the two distributions are distinguished as “wet” and “dry”, the ions in the “dry” distribution still undergo measurable mass loss that indicates that these ions are still hydrated, but less so than the corresponding “wet” distribution.

### Direct observations of ion emission

**Fig. 4:**
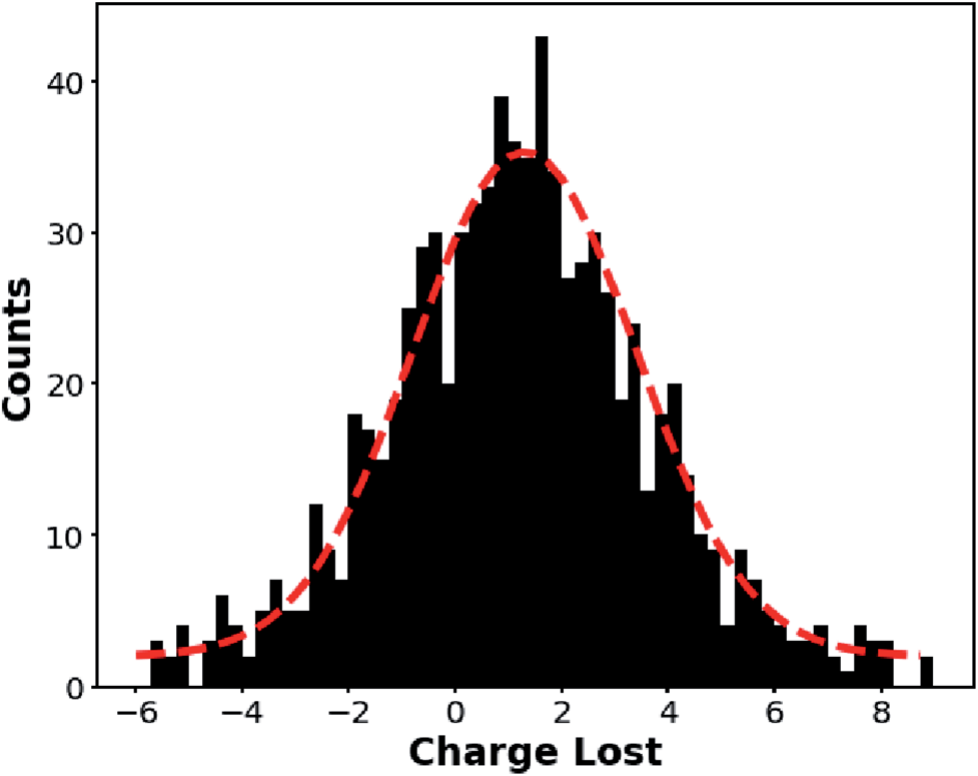
Histogram of the number of charges lost at each ion emission event for individual ions generated from a 20 mM NaCl solution. The observed frequency shifts are exclusively to higher values, indicating that only positively charged ions are emitted. The charge loss distribution is fit by a Gaussian curve (red dotted line) centered at 1.1*e* with standard error of 0.07*e*. The Gaussian fit indicates equal probabilities for a measurement above and below ∼1*e*, meaning that the width of the distribution is primarily a consequence of measurement uncertainty rather than losses of multiple charges.

The sporadic, negative frequency changes observed for many of these ions (Fig. 3, black circles) correspond to charge losses (average number of ion emission events per nanodrop is given in Table 1). The charge before and after each event is determined to obtain the number of charges lost in each event. A histogram of the charge lost in ion emission events for ions formed from 20 mM aqueous NaCl is shown in Fig. 4. Similar histograms for the other solutions for which a significant number of ion emission events were observed (H_2_O, AA, LiCl) are included in the ESI.† Few ion emission events were observed from either KCl or CsCl solutions. Only ions that survive for at least 100 ms before and after the ion emission event without another ion emission event occurring were included in these histograms in order to decrease the charge uncertainty, which is ∼1.8*e* for 100 ms measurements with this CDMS instrument.^47^ The histogram data were fit with a Gaussian function (red dashed line in Fig. 4) and the mean charge loss at each ion emission event for the NaCl data in Fig. 4 is 1.1*e* with a standard error of 0.07*e*. The mean mass loss associated with these charge losses is close to zero, consistent with a minimally solvated ion being emitted from the droplet (average mass loss between 0 and 4 kDa with 2–4 kDa measurement uncertainty: see ESI†). Because charge must be an integer value, we conclude that nearly all the ion emission events correspond to loss of a single charge. Loss (and gain) of a single charge from large analyte ions has been observed previously,^48–50^ but the data reported here represents the first time that ion emission has been directly observed from aqueous nanodrops and in a size range (∼1–10 MDa, ∼15–32 nm diameter) relevant to formation of gaseous macromolecule ions.

The probability an ion emission event for a given charged nanodrop depends on the identity of the solute ion with H_2_O and AA having a discharge rates of ∼0.2 events per ion, whereas KCl and CsCl exhibit few such events (<0.01). The extent of “wetness” or “dryness” does not appear to be directly related to the extent of ion evaporation. For LiCl and NaCl where both “dry” and “wet” ions are observed simultaneously, the rate of charge loss events for these two different types of ions do not appear to be correlated (as indicated in Table 1 and in the Fig. 3 data), suggesting that “dryness” alone does not determine the probability of discharging events. The average masses of nanodrops from different solutions also do not directly correlate to discharge rates (ESI†). For the alkali metal ion solutions, there is a clear trend of decreasing ion emission rate with increasing cation size (Table 1).

### Origins of solution-dependent nanodrop mass and charge

The masses of nanodrops and their corresponding charges depend on the identity of ions present in these aqueous solutions. These results do not appear to be the result of differences in initial droplet sizes produced by electrospray ionization. The conductivity of pure water is lower than that of the ion containing solutions and may lead to differences in initial droplet size. However, the initial droplet sizes for the alkali metal ion containing solutions should be the same, yet the trapped nanodrops are clearly different in mass and charge. Any differences in conductance, vapor pressure, or surface tension in the initial droplets should be minor at 20 mM concentration.^51^ Because the surface tension of the droplets of the different solutions should be approximately the same, Rayleigh fission events for the initial droplets should also be similar at these concentrations. Thus, the differences in the nanodrops that are observed in these experiments must originate from processes that occur after the initial droplet is formed and the first of any Rayleigh fission events that may occur.

The trend in lower charging with increasing cation size for the alkali metal ions follows the trend in ion cation solvation energies where the Gibbs solvation energy of Li^+^ is 575 kJ mol^−1^ relative to the solvation energy of H^+^ (assigned a value of 0 kJ mol^−1^) and is lower than that of Cs^+^ (798 kJ mol^−1^).^52^ H^+^ has the lowest Gibbs solvation energy, and the concentration of this ion in pure water is substantially lower than the positive ions in the other solutions. Both factors should make ion emission less favored for pure water compared to other solutions. Because pure H_2_O nanodrops lack an abundant suitable ion emission “release valve” for excess charge, the formation of more highly charged ions may be kinetically favored leading to “supercharged” nanodrops with charge above the Rayleigh limit (Fig. 2a). This may explain the higher charging observed for micron-sized droplets consisting of volatile solvents,^8^ which evaporate more rapidly than pure water. Another factor that may contribute to the high charging in our experiments is that droplets in vacuum undergo a “freeze-drying” effect where the droplet temperature is significantly lowered by evaporative cooling.^53,54^ This leads to a steady-state internal energy distribution that is substantially below the ambient temperature as the energy lost through evaporative cooling is replenished through gaseous collisions and absorption of blackbody radiation emitted from the surroundings.^55^ The rate of water loss indicates that the temperature of these droplets must be substantially lowered. At sufficiently low temperatures, nanodrops can become ice-like,^11^ which may reduce the rate of ion evaporation. The presence of salts at the nanodrop surface may make it more liquid-like, enabling ion evaporation to occur more readily.

Results for the AA containing nanodrops do not follow the trends in cation solvation energies of the other solutions. The solvation energy of NH_4_^+^ is between that of K^+^ and Rb^+^ yet the charges and masses of the AA containing nanodrops are more similar to that of water than that of KCl. The counterions (acetate *vs.* Cl^−^) differ and may also influence the evaporation process for the cations. Unlike the alkali metal ion salts, AA can undergo proton transfer processes creating a path for their loss as neutrals, which may also lead to the observed differences.

These results provide compelling evidence for the CCRFEM. The observation of ion emission from 15–32 nm nanodrops is consistent with conclusions from prior work which indicated a transition between Rayleigh limited charging and solute ion emission-controlled charging at ∼40 nm.^29^ It is interesting to speculate why charging of proteins and peptides from different solutions have provided limited support for this model. Ions of β-lactoglobulin, bovine ubiquitin and egg-white lysozyme are more highly charged from pure water than from solutions containing alkali metal ions (acetate salts),^32^ consistent with higher charging of aqueous nanodrops. However, the extent of charging of these proteins from solutions containing Li^+^, Na^+^, K^+^, Rb^+^, Cs^+^ as well as NH_4_^+^ are similar.^32^ There is no significant trend in protein ion charging and ion solvation energy except with minimally solvated TMA and TEA.^32^ The significantly lower charge states observed with the latter ions was attributed to collisional dissociation of these positively charged ions from the proteins, a process that occurs to a much lesser extent with the other ions.^32^ Protein conformation also plays a significant role in the extent of charging on proteins.^33^ It is also apparent that the differences in nanodrop charging relative to the Rayleigh limit for these solutions become less distinct when the nanodrop mass is below ∼1 MDa (15 nm diameter). Thus, charging differences predicted by the CCRFEM may not be as applicable to smaller proteins or macromolecular complexes.

### The role of ion evaporation in charging of MDa macromolecular complexes

**Fig. 5:**
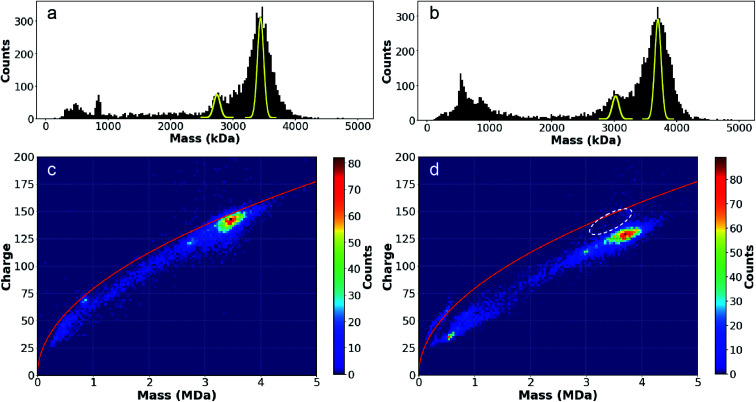
Mass histograms for MS2 capsid ions formed by electrospray from (a) ammonium acetate and (b) NaCl/NaPhos solutions. The yellow peaks correspond to the intrinsic linewidth expected for single ion measurements in this experiment (∼1% of the mass); the broader peak widths observed indicate significant sample heterogeneity due to encapsulation and salt adduction, especially from the NaCl/NaPhos solution. Two-dimensional mass *vs.* charge histograms for MS2 capsid from (c) AA and (d) NaCl/NaPhos show that ions formed from the latter solution are ∼250 kDa higher in mass due to salt adduction, but are charged less (∼13*e*) despite their higher mass (for comparison purposes, the white dotted oval in (d) indicates the AA ion distribution).

The substantial differences in the charging of MDa-sized nanodrops originating from pure water and different salt solutions suggest that these effects may also affect the charging of molecules that have comparable size to the nanodrops investigated here (∼15–32 nm diameters). To investigate if that is the case, an MS2 capsid with a theoretical “empty” mass of 2466 kDa and a diameter of 27 nm ^56^ was analyzed using CDMS from a biochemically preferred solution consisting of 100 mM NaCl and 10 mM Na phosphate (NaCl/NaPhos) and from a 100 mM AA solution commonly used in native mass spectrometry. Source conditions were made more energetic than the nanodrop experiments described above by increasing the voltage between skimmer cones to ∼150 V to desolvate these ions more effectively. The resulting 1D mass histograms and the 2D mass *vs.* charge histograms are shown in Fig. 5. There are two broad high mass peaks in the 1D mass histograms from both solutions (Fig. 5a and b) that are separated by ∼670 kDa. There is an additional, poorly resolved low abundance peak between these two main peaks that is more apparent in the mass histogram from AA (Fig. 5a). A Gaussian fit of the two most abundant peaks from both solutions results in mass values of 2.76 MDa and 3.42 MDa from AA and 3.00 and 3.67 MDa from NaCl/NaPhos. All ions in both solutions that compose these two peaks are “dry”, with no measurable mass change over their lifetimes in the trap. Because the widths of these peaks are much greater than the intrinsic line-width (shown in yellow; based on the ∼1% uncertainty for the measurement of a single ion), each peak must consist of unresolved MS2 capsid species that have encapsulated a range of different cargoes. The native virus RNA (1147 kDa) was not present during the virus assembly. Thus, the heterogeneity observed is almost certainly a result of varied encapsulation of messenger RNA and/or DNA transcripts from the recombinant expression of the capsid, a phenomenon that has been observed previously for MS2 capsids.^56^

The relative abundances of the two major ions from both solutions are similar (Fig. 5a and b). The principal difference between the two mass histograms is that ions formed from the NaCl/NaPhos solution are shifted ∼250 kDa (∼8%) higher in mass and the distributions are wider (∼13% larger standard deviations). The higher mass and wider peak width are almost certainly the result of extensive adduction of non-volatile salts present in the 110 mM NaCl/NaPhos solution. We estimate that the initial droplets formed from a 1.5 μm ESI emitter have an average diameter of roughly 90 nm ^57,58^ and contain ∼1.6 MDa of non-volatile solutes, a substantial fraction of which remain adducted to the gaseous MS2 capsid. This is the first time that MDa-sized macromolecular ions have been analyzed in native MS from a solution containing a 100+ mM concentration of non-volatile salts.

The charge states of the MS2 capsids produced from these two solutions are significantly different. The average charge of MS2 capsid ions formed from the NaCl/NaPhos solution is ∼13 charges (∼10%) lower than the average charge of these ions formed from the AA solution despite the higher masses of the former ions. This can be readily seen from the white oval in Fig. 5d, which shows the range of the higher mass ions formed from AA. This white oval is shifted to lower mass but higher charge. Differing charge-state distributions are most often attributed to conformational changes.^59^ To determine if there are differences in MS2 capsid size indicative of a conformational difference between the two solutions, small angle X-ray scattering (SAXS) experiments were performed.^60^ The SAXS data indicate that the MS2 capsid is slightly more compact in NaCl/NaPhos (0.42 ± 0.93 nm smaller diameter relative to that in AA), representing a ∼3% relative change in the surface area (Fig. S8†). For molecular assemblies with near spherical shape, the extent of charge on the ions formed by electrospray should be proportional to surface area.^18^ Thus, this small difference in diameter does not fully account for the 10% difference in the charge. Dynamic light scattering (DLS) experiments were also performed to verify capsid diameter consistency in both solutions. The DLS data show no detectable capsid size shift between these two buffer systems (Fig. S9†). The effect of any acidification of the solution that may occur during the electrospray process was also explored. DLS data for solutions ranging in pH between 3.5 and 7.5 display no evidence of contraction in capsid diameter in more acidic solutions (Fig. S10†). Thus, it is unlikely that the different charge distributions observed from the two solutions originate from a conformational difference in these two solutions.

The extent of charging of nanodrops with similar physical sizes to that of the MS2 capsid provides insights into the origin of differences of MS2 capsid charging from the two solutions. Aqueous nanodrops with AA are charged to a greater extent than those with NaCl, consistent with the relative extents of charging of the MS2 capsid from the two different solutions. The average charge of the larger, most abundant MS2 species formed from AA solution (Fig. 5c) is 141.5*e*, similar to the value of ∼145*e* for AA containing nanodrops in the same mass range (Fig. 2b). In contrast, the average charge for the MS2 base peak from NaCl/NaPhos (Fig. 5d) is 128.5*e*, which more closely matches the charge of the “dry” distribution of ions produced from the NaCl solution at similar mass (Fig. 2d, ∼120*e*).

### Evidence for solution phase capsid compaction prior to gaseous ion formation

The MS2 capsid is roughly spherical in solution with a diameter of 27 nm.^56^ A 27 nm diameter AA nanodrop has ∼200*e* whereas MS2 ions formed from AA have ∼142*e*. The lower charging of the capsid indicates that the structure collapses to a significant extent in the nanodrop prior to charging of the gaseous ion. An estimate of the MS2 capsid diameter of 21.8 nm is obtained from AA nanodrops with the same charge. Similar values obtained through differential ion mobility have been reported for gaseous MS2 ions.^61–63^ The apparent capsid density of 1.04 g mL^−1^ is substantially less than that of an average protein (1.35 g mL^−1^)^64^ or RNA/DNA (∼1.7 g mL^−1^),^65^ suggesting that this compaction is not complete. The extent to which native macromolecular structure is preserved in the gas phase is a matter of ongoing debate.^30,66^ Ion mobility measurements have shown that large protein complexes, such as GroEl, can undergo a “gas-phase collapse” to produce a compacted structure.^30^ This compaction is generally assumed to take place after gaseous ion formation.^67–69^ Our data indicate that the structure of MS2 capsid is compacted by ∼5 nm in diameter relative to its solution-phase structure. Moreover, because the charge state is determined at the final stages of droplet evaporation, our charge measurements show that this compaction likely occurred in the droplet at or before this stage. It is plausible that compaction followed by ion evaporation from the desolvated ion may also contribute to the lower observed charge states,^50^ although ion evaporation from the desolvated ions was not observed in our experiments.

## Conclusions

The masses and charges of nanodrop ions generated from pure H_2_O and 20 mM aqueous solutions of AA, LiCl, NaCl, KCl, and CsCl were measured on an ion-by-ion basis using CDMS. Nanodrop sizes ranging from 1–10 MDa (15–32 nm diameter) are trapped and the mass and charge evolution of individual ions show that two distinct types of ions were generated. “Wet” ions from the H_2_O, AA, LiCl, and NaCl solutions, exhibited significant mass loss corresponding to evaporation of thousands of water molecules over the 1 s trapping time whereas “dry” ions from LiCl, NaCl, KCl, and CsCl solutions lost little mass. Ion emission events corresponding to the loss of a singly charged minimally solvated ion occurs for both “wet” and “dry” ions. This is the first direct observation of ion emission from aqueous nanodrops in the nanometer size range. These results provide compelling evidence for the CCRFEM for charging of MDa+ macromolecular complexes in native mass spectrometry. Charging of the nanodrops depends on the identity of charge carriers in solution. There is clear trend of lower nanodrop mass and charge with increasing alkali metal cation size, and these results also indicate that solute identity affects droplet evaporation after the initial formation of the charged droplets.

The mass and charge distribution of MS2 capsid ions from AA typically used in native MS differs from those formed from a biochemically preferred NaCl/NaPhos solution containing 100+ mM of non-volatile solutes. Ions formed from NaCl/NaPhos have ∼8% higher masses as a result of extensive adduction, but significantly lower charge, consistent with ion evaporation of Na^+^ lowering the charge on the droplets from which these ions are formed. The extent of MS2 capsid charging from the AA solution indicates that the structure partially collapses in the nanodrops prior to when charging of the gaseous ions occurs. Ion mobility results have shown that compaction of other macromolecular assemblies occur,^30^ but our results localize this compaction to a time prior to gaseous ion formation. This is the first time MDa-sized ions have been analyzed in native MS from a biochemically relevant solution. Future work on calibrating the mass shift based on peak widths, analogous to what has been done for much smaller complexes,^70^ could make this a practical method to obtain accurate masses of large complexes from a wide range of buffers that contain nonvolatile salts typically used in biochemistry.

## Conflicts of interest

There are no conflicts to declare.

## Supplementary Material

SC-012-D0SC05707J-s001

SC-012-D0SC05707J-s002

SC-012-D0SC05707J-s003

SC-012-D0SC05707J-s004

SC-012-D0SC05707J-s005

SC-012-D0SC05707J-s006
