# Supplementary figures and images for: Direct observation of ion emission from charged aqueous nanodrops: effects on gaseous macromolecular charging
Source: Chem Sci. 2021 Feb 27;12(14):5185–95. doi: 10.1039/d0sc05707j (PMC8179642; doi:10.1039/d0sc05707j)

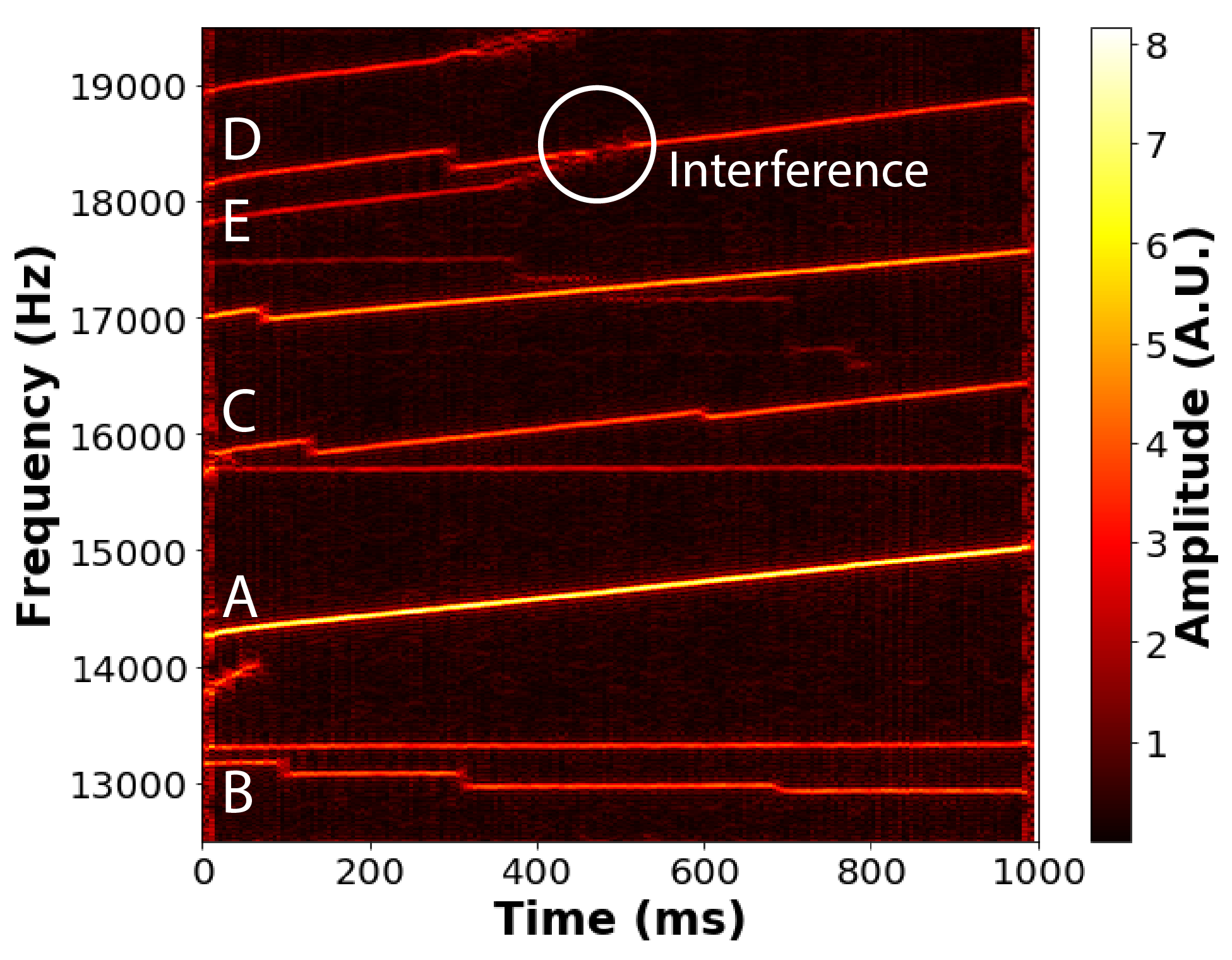

Supplement: SC-012-D0SC05707J-s001 [file SC-012-D0SC05707J-s001.tif]

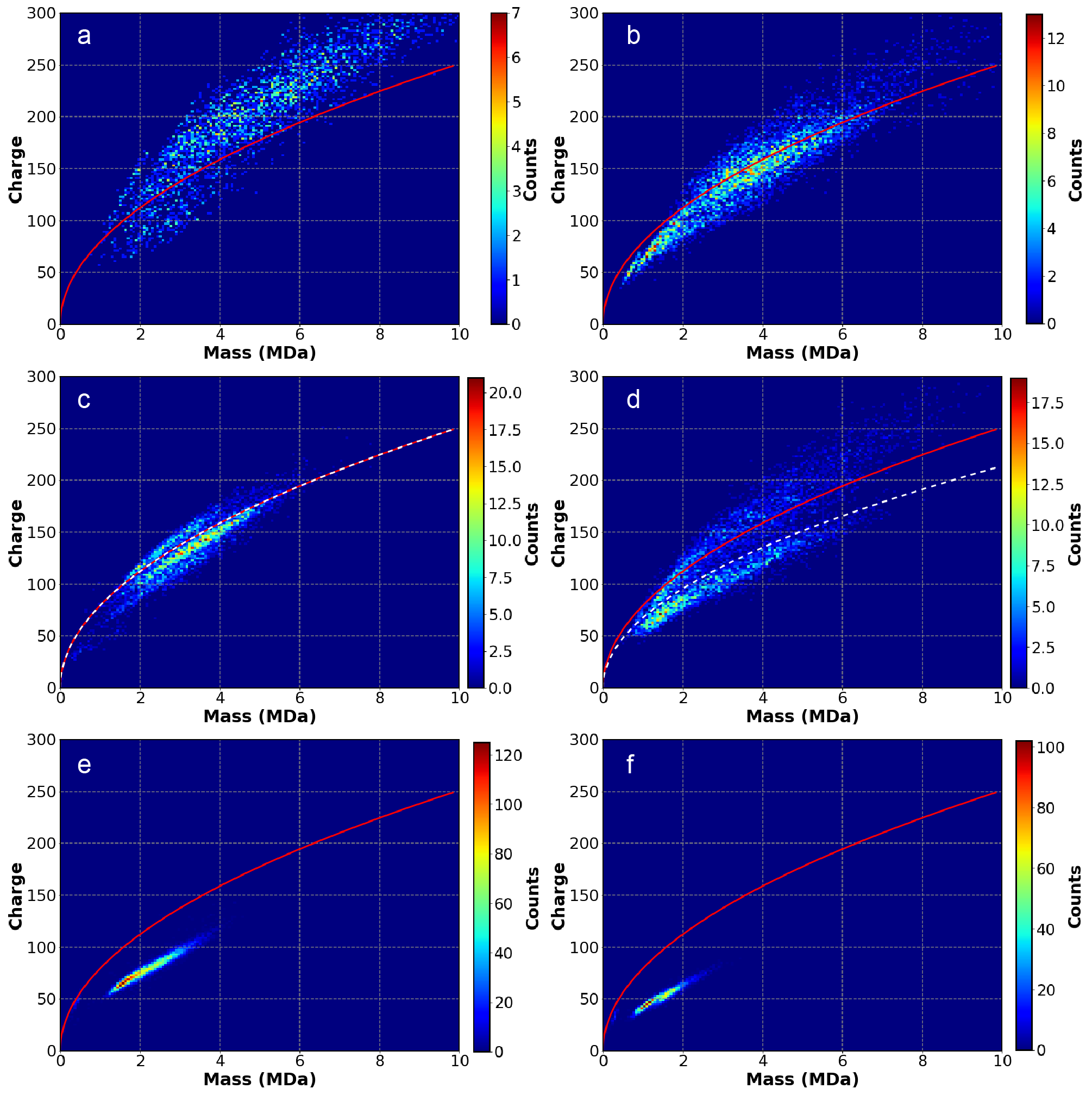

Supplement: SC-012-D0SC05707J-s002 [file SC-012-D0SC05707J-s002.tif]

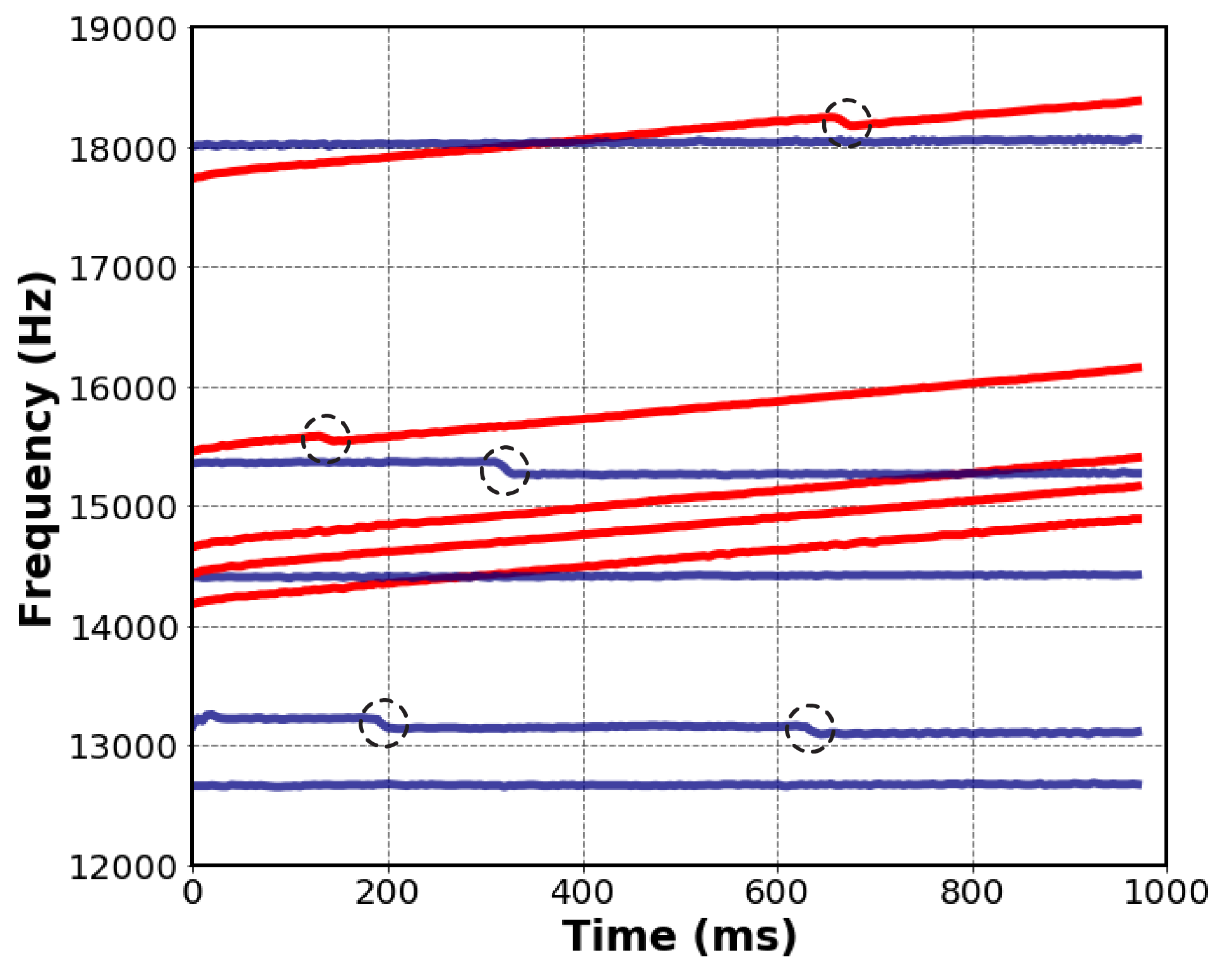

Supplement: SC-012-D0SC05707J-s003 [file SC-012-D0SC05707J-s003.tif]

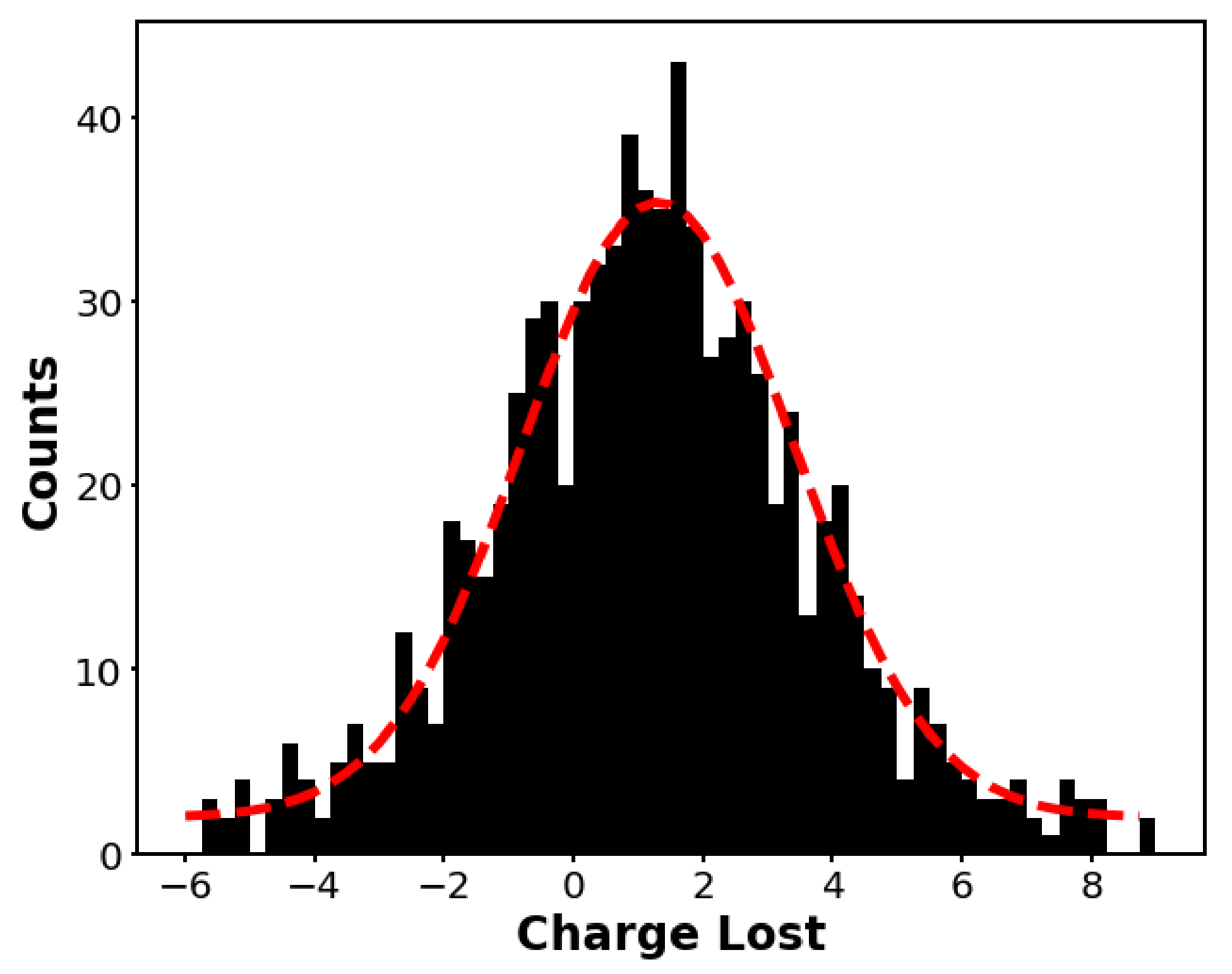

Supplement: SC-012-D0SC05707J-s004 [file SC-012-D0SC05707J-s004.tif]

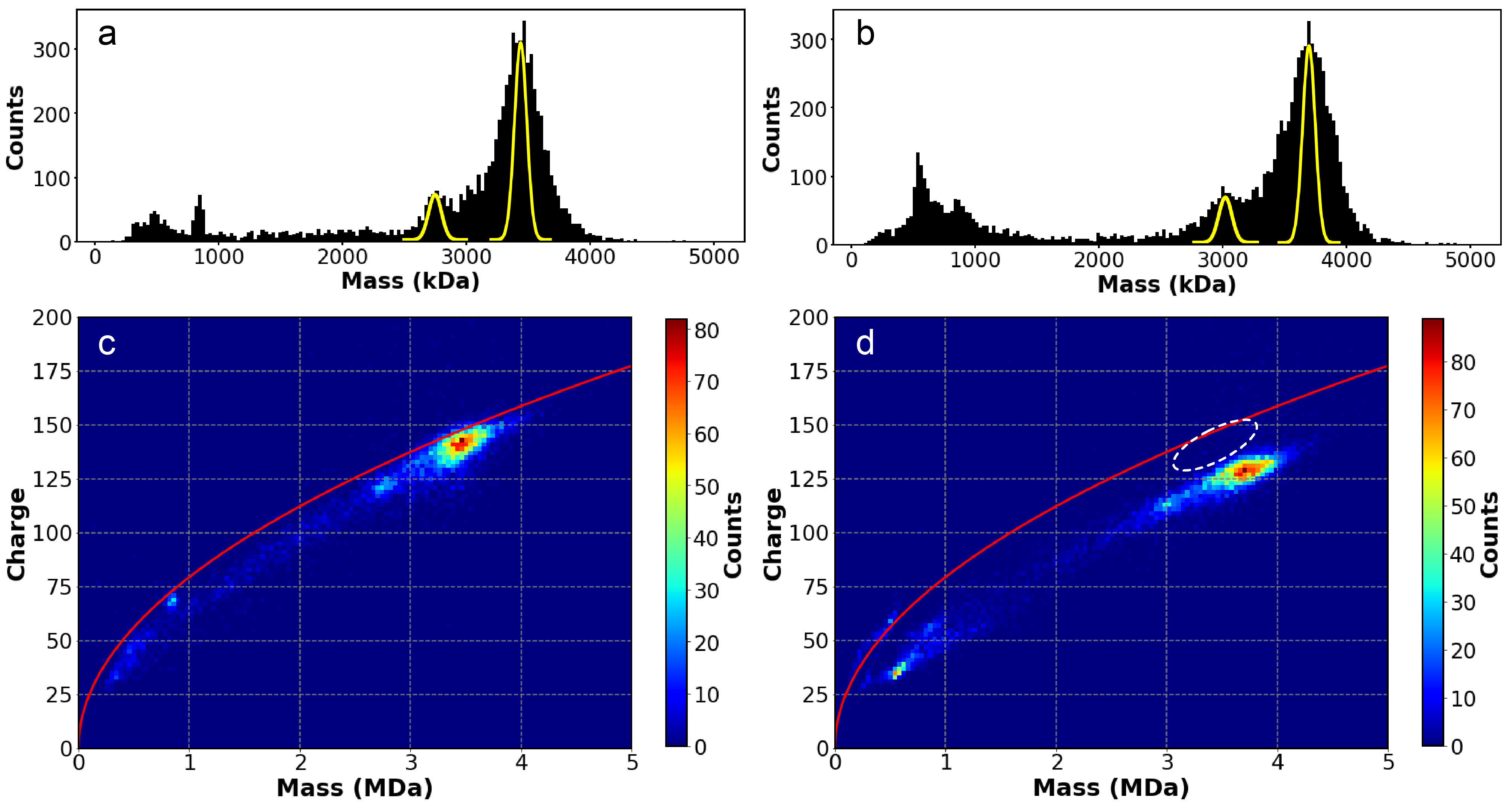

Supplement: SC-012-D0SC05707J-s005 [file SC-012-D0SC05707J-s005.tif]
