# Supplementary material for: Direct observation of ion emission from charged aqueous nanodrops: effects on gaseous macromolecular charging
Source: Chem Sci. 2021 Feb 27;12(14):5185–95. doi: 10.1039/d0sc05707j (PMC8179642; doi:10.1039/d0sc05707j)
Supplement: SC-012-D0SC05707J-s006 [file SC-012-D0SC05707J-s006.docx]

**Supporting Information:**

**Direct Observation of Ion Emission from Charged Aqueous Nanodrops: Effects on Gaseous Macromolecular Charging**

Conner C. Harper, Daniel D. Brauer, Matthew B. Francis, and Evan R. Williams*

*Department of Chemistry, University of California, Berkeley, California, 94720-1460*

For submission to Chemical Science

^*^Address correspondence to this author.

Email: erw@berkeley.edu

**Experimental Details: Charge Detection Mass Spectrometry.** Ions are introduced into the CDMS instrument through a modified Waters Z-Spray source (Waters Corporation, Milford, MA) and enter a region containing two RF-only quadrupole ion guides (Ardara Technologies, Ardara, PA) at a pressure of ~1 × 10^-3^ Torr. The DC potential of the first guide is ~10 V higher than that of the second guide. Ions with *m*/*z* below ~10,000 are lost in this stage due to the low mass cut-off of the RF quadrupole ion guides operating at 555 kHz. A lens at the exit of the ion guides with a potential ~80 V higher than that of the second ion guide is used to accumulate ions for up to 1 s. The voltage on this lens is pulsed down by 110 V for 1 ms, which introduces a pulse of ions into a turning quadrupole (Ardara Technologies, Ardara, PA) that directs the ions into the high vacuum chamber containing an electrostatic cone trap and detector tube. The pressure in the final chamber was ~4 × 10^-9^ Torr. The potential of the entrance cone electrode is lowered to 0.0 V for 1 ms with a 500 μs delay relative to the release of the ions from the RF quadrupole to allow for ions to transit from the RF trap into the electrostatic ion trap. The potential is then raised to match the end cone (330 V) for the duration of the 1 s trapping event. Ions in the electrostatic ion trap induce a charge pulse each time they traverse the detector tube. Individual ion masses, charges, and energies are determined at each 5 ms increment from the measured oscillation frequencies and amplitudes of the fundamental and second harmonic frequencies as they evolve throughout the 1 s trapping period. The broad distribution of ion mass and charge in these experiments makes it possible to simultaneously analyze up to ~40 individual ions in each trapping event because the frequency distribution of the ions is broad (up to 10 kHz) and their signals are less likely to interfere with each other. Ion energies are centered around 211 eV/z with a standard deviation of 5 eV/z. The induced charge signal is amplified by a CoolFET charge-sensitive preamplifier (Amptek) and home-built linear voltage and current amplifier. The signal is digitized and analyzed with a Python program using short-time Fourier transforms (STFT) and a peak-picking algorithm. Interfering ion signals and ion signals that do not persist for the entire 1 s trapping period are discarded.

**Charge and Mass Lost after Ion Emission Events.** Figures S1–S7 show charge and mass loss histograms as a result of ion emission events for nanodrops originating from pure water (S1-S2), ammonium acetate (S3–S4), LiCl (S5–S6), and NaCL (S7, mass loss only). In general, Gaussian fits of charge losses are centered near 1 *e* and fits of mass losses are centered near 0 Da within measurement error. These data are consistent with the loss of a singly charged ion that is minimally solvated.

**Experimental Details: Small Angle X-ray Scattering (SAXS).** MS2 capsid samples were prepared in triplicate at a range of concentrations between 50 uM and 6.7 uM in 100 mM ammonium acetate at pH 7.2 or 10 mM sodium phosphate buffer at pH 7.2 with 100 mM NaCl. Samples were analyzed by high-throughput small angle X-ray scattering (HT-SAXS) at the SIBYLS beamline at the Advanced Light Source in Berkeley CA. Samples were exposed with a 10^11^ photon/s, 12 keV monochromatic beam in a series of exposures: 0.5, 1.0, 2.0, and 4.0 s. Background subtraction was performed using measurements of buffer-only samples. Data were first analyzed using the SAXS FrameSlice web app and then imported into the ScÅtter and SASview applications for further size and shape analysis. Data were fit to a polydisperse core-shell sphere model using a fixed protein shell scattering length density as described in previous MS2 SAXS measurements.^1^

**Experimental Details: Dynamic Light Scattering (DLS).** DLS measurement was performed on a Malvern Instruments Zetasizer Nano ZS. MS2 capsid samples were prepared at a protein concentration of 10 uM in each respective buffer and passed through a 0.2 μM spin filter prior to each run. Size determination was performed in triplicate at 25 ºC following a 2-min temperature equilibration.

**Correlation of Nanodrop Mass and Solute Identity.** A notable result from these nanodrop experiments is that the average mass of the nanodrops generally correlates with the solvation energies of the charge carriers in solutions (as seen in Table 1). Electrospray conditions were held approximately constant between solutions, so it is unlikely that the large mass differences observed are the merely the result of electrospray variations. While this correlation is still under investigation, these data suggest that less solvated charge carriers may promote the formation of either smaller Rayleigh fission progeny droplets or accelerate the droplet evaporation process by some other means. Early stage differences in droplet evaporation may also amplify the observed differences in nanodrop size, as the increased ratio of surface area to volume of smaller droplets, among other factors, is conducive to faster evaporation rates.

**References**

1 D. A. Kuzmanovic, I. Elashvili, C. O’Connell and S. Krueger, *Radiat. Phys. Chem.*, 2008, **77**, 215–224.


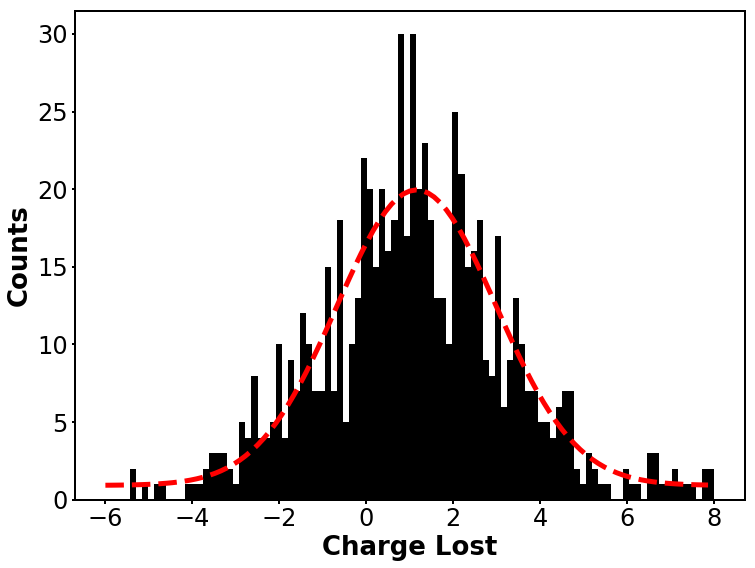
**Figure S1.** Histogram of the number of charges lost at each ion emission event for individual ions generated from pure water. The charge loss distribution is fit by a Gaussian curve (red dotted line) centered at 1.01 *e* with standard error of 0.07 *e*.

**
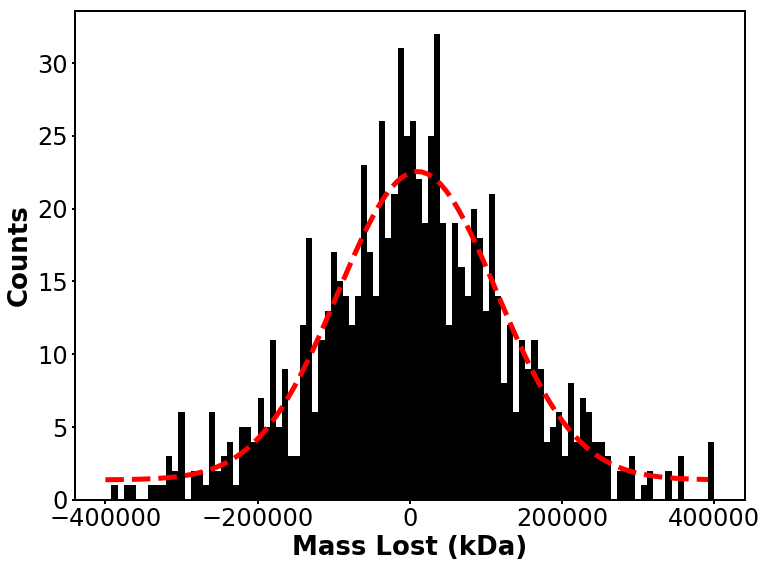
**

**Figure S2.** Histogram of the mass lost at each ion emission event for individual ions generated from pure water. The mass loss distribution is fit by a Gaussian curve (red dotted line) centered at 2,000 Da with standard error of 4,000 Da.


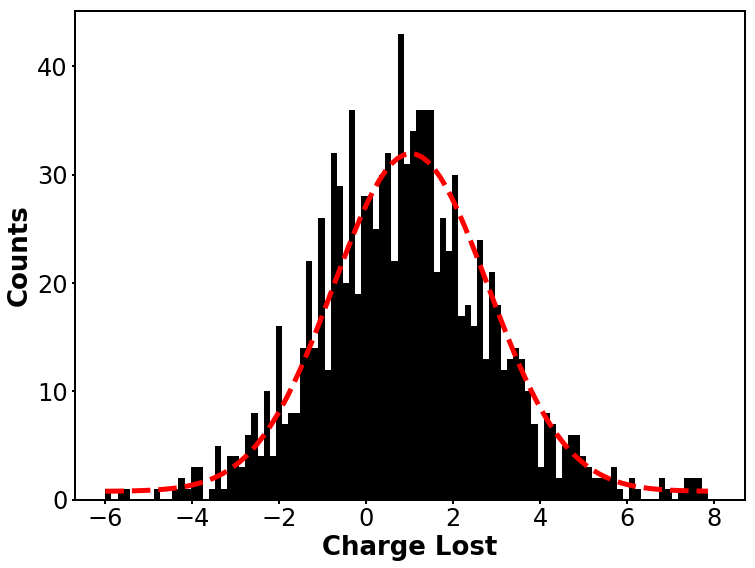


**Figure S3.** Histogram of the number of charges lost at each ion emission event for individual ions generated from 20 mM ammonium acetate. The charge loss distribution is fit by a Gaussian curve (red dotted line) centered at 1.03 *e* with standard error of 0.05 *e*.


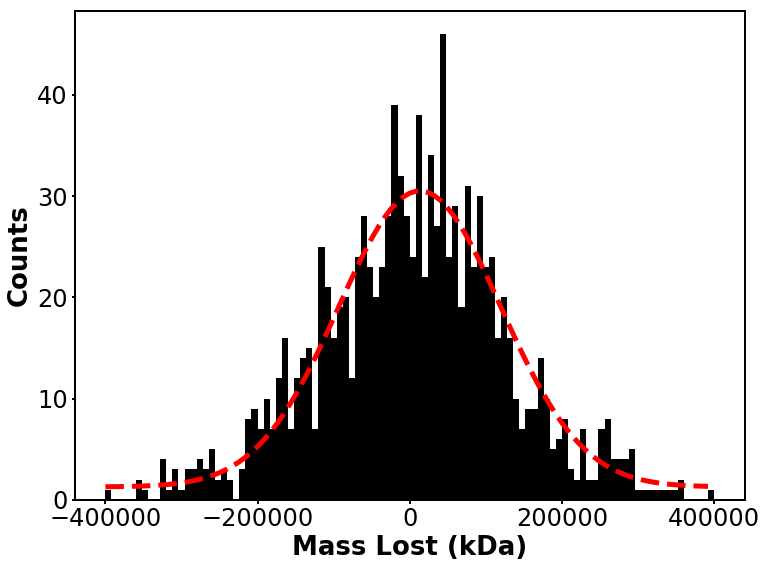


**Figure S4.** Histogram of the mass lost at each ion emission event for individual ions generated from 20 mM ammonium acetate. The mass loss distribution is fit by a Gaussian curve (red dotted line) centered at 4,000 Da with standard error of 3,000 Da.


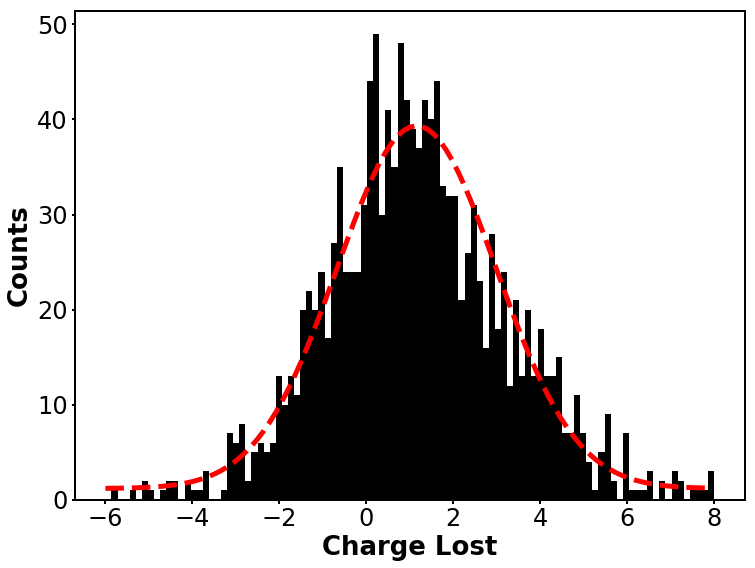


**Figure S5.** Histogram of the number of charges lost at each ion emission event for individual ions generated from 20 mM LiCl. The charge loss distribution is fit by a Gaussian curve (red dotted line) centered at 0.97 *e* with standard error of 0.05 *e*.

**
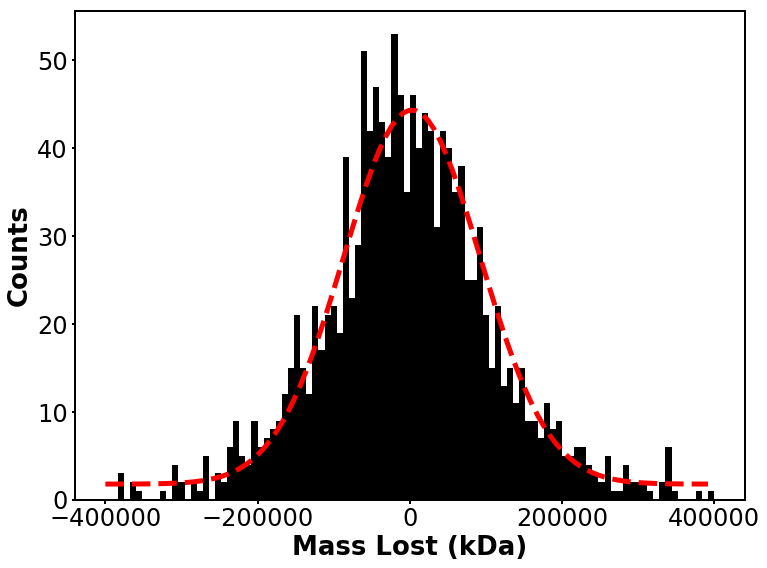
**

**Figure S6.** Histogram of the mass lost at each ion emission event for individual ions generated from 20 mM LiCl. The mass loss distribution is fit by a Gaussian curve (red dotted line) centered at 0 Da with standard error of 2,000 Da.


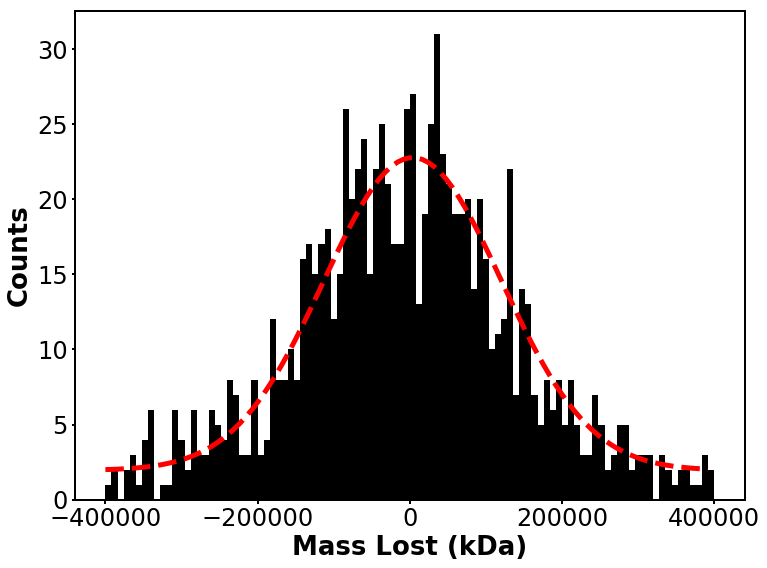


**Figure S7.** Histogram of the mass lost at each ion emission event for individual ions generated from 20 mM NaCl. The mass loss distribution is fit by a Gaussian curve (red dotted line) centered at 1,000 Da with standard error of 4,000 Da.

**Figure S8.** Correlation plot of the average mass of droplets observed from each solution and ion emission rate.


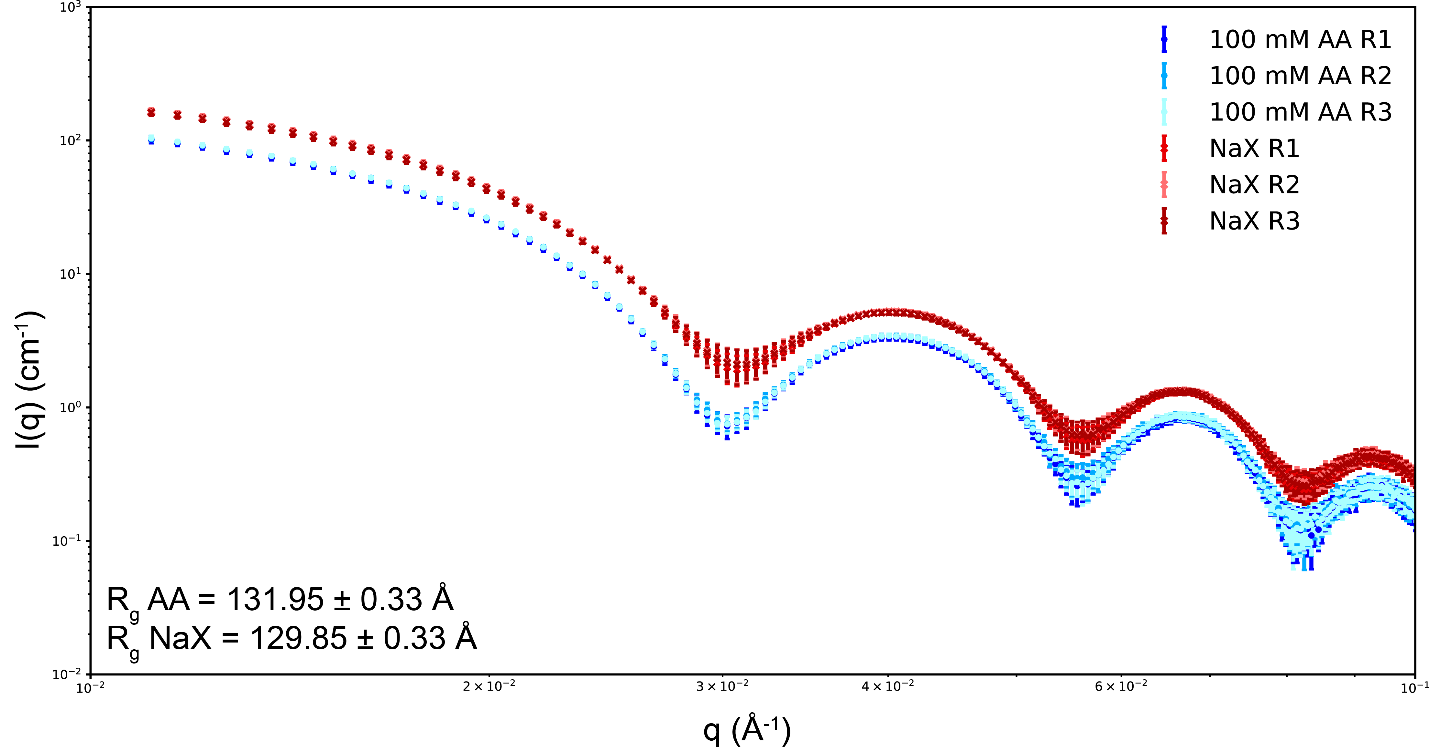


**Figure S9.** Intensity vs q profile of MS2 capsids from SAXS in AA (100 mM ammonium acetate) or NaX (10 mM sodium phosphate + 100 mM NaCl) buffer. The mean radius of gyration (R_g_) was determined by fit to a core-shell sphere model and reported as the sum of the core and protein shell radii.


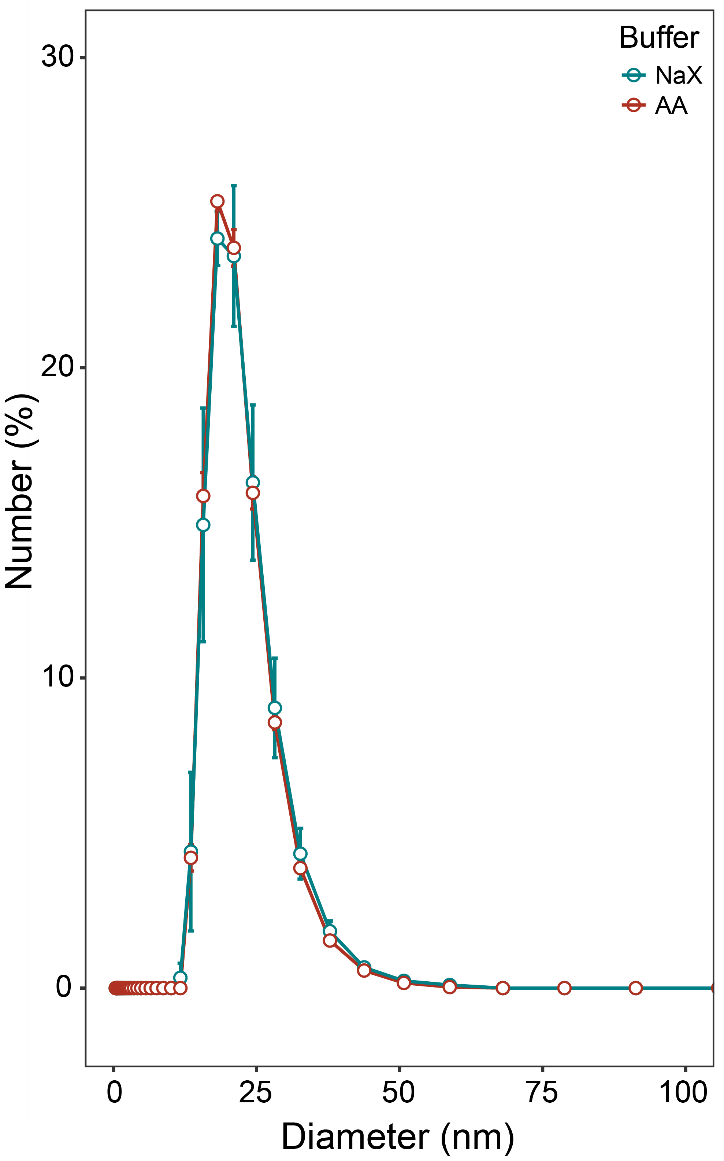


**Figure S10.** MS2 capsid diameter in NaX or AA buffer as determined by DLS. Size distribution is reported as percent number of particles. Error bars represent the standard deviation of three replicate measurements.


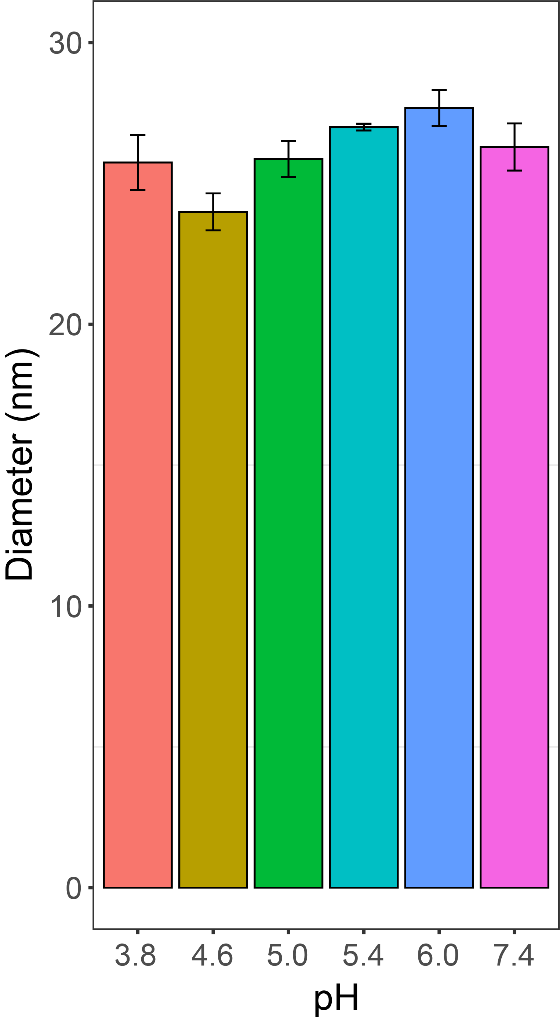


**Figure S11.** Effect of pH on MS2 capsid diameter as determined by DLS. Samples were prepared in 10 mM sodium phosphate buffer with 100 mM NaCl at the indicated pH. Error bars represent the standard deviation of three replicate measurements.
